# Lipid-Binding Regions within PKC-Related Serine/Threonine Protein Kinase N1 (PKN1) Required for Its Regulation

**DOI:** 10.1021/acs.biochem.4c00009

**Published:** 2024-03-05

**Authors:** Jason L. J. Lin, Hanna S. Yuan

**Affiliations:** † Genomics Research Center, Academia Sinica, Taipei 11529, Taiwan; ‡ Institute of Molecular Biology, Academia Sinica, Taipei 11529, Taiwan; § Department of Biochemistry and Molecular Biology, University of Melbourne, Victoria 3010, Australia

## Abstract

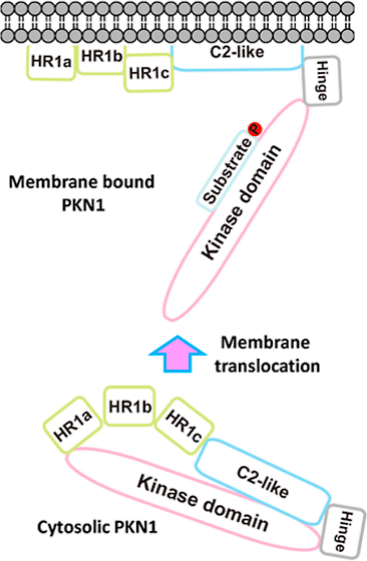

PKC-related serine/threonine protein kinase N1 (PKN1) is a protease/lipid-activated protein kinase that acts downstream of the RhoA and Rac1 pathways. PKN1 comprises unique regulatory, hinge region, and PKC homologous catalytic domains. The regulatory domain harbors two homologous regions, i.e., HR1 and C2-like. HR1 consists of three heptad repeats (HR1a, HR1b, and HR1c), with PKN1-(HR1a) hosting an amphipathic high-affinity cardiolipin-binding site for phospholipid interactions. Cardiolipin and C18:1 oleic acid are the most potent lipid activators of PKN1. PKN1-(C2) contains a pseudosubstrate sequence overlapping that of C20:4 arachidonic acid. However, the cardiolipin-binding site(s) within PKN1-(C2) and the respective binding properties remain unclear. Herein, we reveal (i) that the primary PKN1-(C2) sequence contains conserved amphipathic cardiolipin-binding motif(s); (ii) that trimeric PKN1-(C2) predominantly adopts a β-stranded conformation; (iii) that two distinct types of cardiolipin (or phosphatidic acid) binding occur, with the hydrophobic component playing a key role at higher salt levels; (iv) the multiplicity of C18 fatty acid binding to PKN1-(C2); and (v) the relevance of our lipid-binding parameters for PKN1-(C2) in terms of kinetic parameters previously determined for the full-length PKN1 enzyme. Thus, our discoveries create opportunities to design specific mammalian cell inhibitors that disrupt the localization of membrane-associated PKN1 signaling molecules.

## Introduction

The PKC family of serine/threonine kinases comprises classical, novel, and atypical PKC subfamilies.^1,2^ The classical (or conventional) PKC (cPKC) is the best-studied kinase, which harbors pseudosubstrate, C1a, C1b, C2, and catalytic domains. In 2011, a combined X-ray crystallographic and small-angle X-ray scattering analysis reported the structure of full-length autoinhibited cPKC βII.^3^ This structure reveals that the cPKC pseudosubstrate motif is located N-terminal to its C1a and C1b domains and can block access for the PKC target substrate binding. Moreover, it has been shown that the C2 domain is responsible for the initial membrane association of cPKC that facilitates binding between C1a or C1b and activating diacyl glycerol (DAG), thereby eliciting a catalytically competent conformational status for cPKC.^3^ Interestingly, novel PKC (nPKC) features a C2-like domain located N-terminal to its C1a, C1b, and pseudosubstrate domains, suggesting that nPKC exerts a distinct regulatory function to that of cPKC.

**Figure 1:**
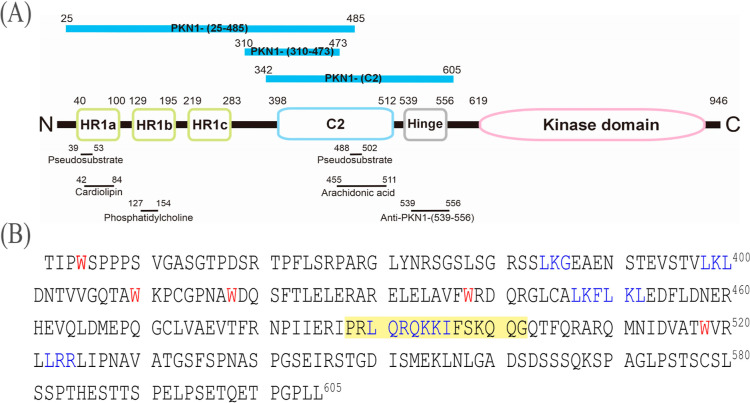
Primary structure of rat PKN1. (A) PKN1 consists of a unique regulatory domain comprising HR1 featuring three heptad repeats (HR1a, HR1b, and HR1c) and a C2-like domain, a hinge region, a PKC homologous kinase (catalytic) domain, and a short C-terminal extension.^5^ PKN1-(HR1a) contains a pseudosubstrate motif that overlaps with an amphipathic cardiolipin-binding (high-affinity) site. A phosphatidylcholine-binding site was also identified within PKN1-(HR1b). Three PKN1 constructs including PKN1-(C2)-(342–605), the focus of this study, PKN1-(310–473), and PKN1-(25–485) were expressed as recombinant proteins. A conserved nPKC C2-like pseudosubstrate motif [PKN1-(488–502)] has been identified [highlighted in yellow in (B)] as overlapping with the arachidonic acid (C20:4) binding region, i.e., [PKN1-(455–511)]. An antibody raised against the hinge region [PKN1-(539–556)] was used for immunostaining analysis. (B) Five tryptophan residues in PKN1-(C2), i.e., Trp^345^, Trp^410^, Trp^418^, Trp^438^, and Trp^518^, served as fluorophores (red letters) for intrinsic fluorescence spectrometry analysis. The PKN1-(C2) pseudosubstrate sequence is highlighted in yellow. Conserved amphipathic L(I)R(K)X-like motifs (blue letters), similar to those present in the HR1 domain, are also present in the PKN1-(C2) sequence.

The protein kinase C-related kinases (PKNs; PKN1, PKN2, and PKN3) represent additional members of the PKC family.^4−7^ Unlike the cPKCs, the substrate kinase activity of PKN1 is more similar to that of the nPKCs, being unresponsive to the presence of calcium ions.^8−10^ PKNs interact with small GTP-binding proteins such as RhoA, RhoB, RhoC, or Rac1 to regulate assembly of focal adhesion and actin stress fibers.^11−15^ PKNs are activated by unsaturated C18–C20 fatty acids and acidic phospholipids such as cardiolipin.^10,16^ Moreover, they are known to protect neurons and promote the survival of cardiac myocytes under stressed conditions.^17,18^ Notably, high expression levels of PKN1 have been detected in human prostate and ovarian cancer cells,^19,20^ seemingly promoting their growth and development.^21^ Structurally, PKN1 has an N-terminal regulatory domain, a hinge region, a kinase (catalytic) domain, and a C-terminal variable region (Figure 1A).^5,22^

The N-terminal regulatory domain contains two homologous regions (HR1 and C2-like), with the former comprising three heptad repeats (HR1a, b, and c) arranged as antiparallel helices.^5,13,14,23^ Crystal structure analyses of human PKN1 have revealed that its residues 40–92 [HR1a-(40–92)] that can interface with a RhoA GTPase molecule overlap with the higher-affinity cardiolipin-binding site and the pseudosubstrate HR1a-(39–53) sequence (Figure 1A).^5,13,24,25^ More recently, a conserved phosphatidylcholine-binding motif was identified within the HR1b subdomain (Figure 1A).^24^ Hence, it is likely that binding between the HR1a and b subdomains with phospholipids represents the leading event(s), followed by RhoA interaction, for PKN1 activation. PKN1 [C2-(398–512)] in rat corresponds to the nPKC C2-like domain.^5^ An X-ray crystallographic study indicated that the C2-like domain of nPKCε adopts an antiparallel β-stranded conformation (Figure S1B).^26^ Similar to the C2-like domain of nPKCs, PKN1 hosts a pseudosubstrate sequence [C2-(488–502)] that overlaps with the arachidonic acid-binding sequence [C2-(455–511)]^5,27,28^ (Figure 1). Cardiolipin is by far the most potent lipid activator of PKN1.^8,10^ In the bovine heart, C18 fatty acids represent up to 95% of the cardiolipin acyl composition (Table S2). Oleic acid has been identified as a significantly more effective C18 fatty acid activator of PKN1 compared to other tested fatty acids.^10^ Hence, we were interested in determining the molecular mechanism underlying the interactions between C18 fatty acids and PKN1-(HR1) or -(C2). Recent identification of the high-affinity cardiolipin-binding sites (*K*_d_ < 150 nM) located within the HR1a subdomain has provided a structural basis for comparative analysis of the C2-like domain^5,24^ (Figure 1). Five potential cardiolipin-binding L(I)R(K)X-like motifs, similar to those present in the HR1 domain sequences, occur within PKN1-(C2) (Figure 1B). Hence, we had three principal objectives in this study: (i) to characterize the structures of PKN1-(C2); (ii) to measure the binding parameter(s) between PKN1-(C2) and phospholipids or fatty acids; and (iii) to investigate the molecular mechanism underpinning PKN1-(C2) and lipid interactions. Our results provide new structural and functional insights into how PKN1 is activated and regulated by phospholipids and fatty acids.

## Materials and Methods

### Reagents

All reagents used were of analytical grade and obtained commercially, as described previously.^24^ The polyclonal anti-PKN1-(539–556) antibody was raised against amino acids 539–556 within the hinge region of rat liver PKN1 (Dr. Wettenhall, University of Melbourne, Australia). Both rabbit monoclonal anti-PKN1 (ab195264) and goat polyclonal antirabbit IgG-HRP (ab97051) (Abcam, United Kingdom) were acquired commercially. All lipid preparations were purchased from Sigma-Aldrich, and relevant technical information is presented in Table S2.

### Recombinant Constructs and Bacterial Expression

Recombinant rat PKN1-(HR1) was generated as described previously.^24^ PKN1-(C2)-(342–605) was amplified by polymerase chain reaction (PCR) from rat liver using the primers: 5′-AAACCTTCATATGACCATCCCTTGGAGCCCTCCC-3′ (forward) and 5′-GGGGACTCTCGAGTAGAGGGCCTGGGGTCTCCTG-3′ (reverse). The PCR-amplified fragments were cloned between NdeI and XhoI sites of the expression vector pET15b (Novagen) to generate the 6x histidine-tagged recombinant protein. The in-frame sequences of both constructs were confirmed by automated DNA sequencing using the ABI Prism Big Dye Terminator cycle sequencing ready reaction kit (PerkinElmer). The pET15b-PKN1-HR1-(342–605) construct was transformed into *Escherichia coli* strain BL21 (DE3) λ to express the recombinant proteins. The pET15b-PKN1-(310–473) and pET15b-PKN1-(25–485) plasmids were synthesized commercially (Genomics BioSci. & Tech., Taiwan) and transformed into *E. coli* strain HIT-21 (RBC Bioscience).

### Purification of Recombinant PKN1-(HR1), PKN1-(C2), and PKN1-(310–473)

Purification of PKN1-(HR1), PKN1-(C2), and PKN1-(310–473) under denaturing conditions was conducted according to a protocol described previously.^29^ The metal ion affinity-purified recombinant PKN1-(HR1) and PKN1-(C2) proteins were dialyzed against a buffer of 30 mM HEPES pH 7.4, 0.3 M NaCl, 3 mM reduced glutathione, 5% (v/v) glycerol, 5 mM glycine, 0.005% (v/v) Tween 20, and 0.5 mM phenylmethanesulfonylfluoride (PMSF) at 4 °C for 16 h. The dialysates were further dialyzed in 20 mM phosphate buffer (Na_2_HPO_4_ + NaH_2_PO_4_) pH 7.4, 25 mM NaCl, and 0.5 mM PMSF at 4 °C for 12 h. PKN1-(310–473) was dialyzed against 30 mM HEPES pH 7.4, 0.3 M NaCl, 3 mM reduced glutathione, 5% (v/v) glycerol, 5 mM glycine, and 0.5% (v/v) Brij-53 at 4 °C for 16 h. Protein concentrations of the dialysate fractions were determined, and the proteins were stored at −70 °C until further use.

### Bacterial Expression and Purification of the Recombinant PKN1-(25–485)

One single colony of fresh transformants containing pET15b-PKN1-(25–485) was inoculated for growth overnight at 37 °C in a Luria broth medium supplemented with ampicillin (100 μg/mL). The cells were grown at 37 °C for 41/2 h, and when the OD_600nm_ reached 0.6, 0.8 mM isopropyl β-d-1-thiogalactopyranoside was added and the culture continued to grow at 18 °C for 20 h. The cells were harvested at 15,000 rpm and frozen at −80 °C. The cells were thawed and fully resuspended in buffer [1× PBS pH 8, 10% glycerol, 10 mM imidazole, and 0.2% 5-cyclohexyl-1-pentyl-β-d-maltoside (Cymal-5)], followed by cell disruption using a microfluidizer (M-110L, Microfluidics Corp., USA). The disrupted cells were incubated at 25 °C for 1 h, followed by centrifugation at 15,000 rpm, before subjecting the supernatant to metal ion affinity chromatography and washing with a buffer of 1× PBS pH 8, 10% glycerol, and 10 mM imidazole. The affinity-bound target protein was eluted using the same buffer supplemented with 500 mM imidazole and 0.2% Cymal-5.

### Size-Exclusion Chromatography of PKN1-(HR1), PKN1-(C2), and PKN1-(25–485)

The PKN1-(HR1) or PKN1-(C2) proteins were separately applied to a 25 mL Sephacryl 200 (S-200) size-exclusion column pre-equilibrated in a buffer containing 20 mM phosphate buffer pH 7.4 and 150 mM NaCl at 4 °C. The column was calibrated as described previously.^24^ The affinity-purified PKN1-(25–485) was applied to the Superdex-200 Increase (10/300 GL) column in a buffer of 50 mM Tris-HCl pH7.5, 150 mM NaCl, and 0.075% Brij-35, and then the peak fractions collected were pooled. All protein concentrations were determined prior to storage at −70 °C until further use.

### Circular Dichroism Spectroscopy

The UV circular dichroism (CD) spectra of PKN1-(C2) were recorded at 25 °C on an AVIV 62DS CD spectrophotometer (Lakewood, NJ, USA) in the range of 197–248 nm using a quartz cell with a 1 mm light path. The spectra were recorded as an average of five scans for each recombinant protein sample after correction for the baseline contributions of buffer and cardiolipin. The data are reported as mean residue molar ellipticities ([θ]) or deg × cm^2^/mol. The protein secondary structure from CD spectra was calculated by using the PROSEC 3.0 program (AVIV, USA).

### Protein Tryptophan Emission Fluorescence Spectrometry

Intrinsic tryptophan fluorescence was measured using LS-5 luminescence (PerkinElmer). Intrinsic fluorescence of PKN1-(C2) tryptophan residues (Trp^345^, Trp^410^, Trp^418^, Trp^438^, and Trp^518^) or Trp^13^ of PKN1-(HR1) was used to monitor structures of the peptides in solution. The spectra were obtained by using an excitation wavelength of 296 nm and emission scans at 0.5 nm intervals from 310 to 410 nm. The 2 mL quartz cuvettes used in these experiments were pretreated with dichlorodimethylsilane to minimize the loss of peptides due to adsorption.

### Preparation of the Phospholipid Vesicles

The phospholipids were dissolved in analytical-grade methanol–chloroform solvent (1:1, v/v), and the phospholipid stock solutions were stored at −20 °C at a concentration range of 4–20 mM. Prior to use in binding studies, aliquots of the phospholipid stock solutions were dried under a stream of N_2_ gas, and the phospholipids were resuspended in 20 mM phosphate buffer pH 7.4 and 25 mM NaCl at room temperature. The resuspended phospholipid preparations were sonicated for 3 min to produce small unilamellar vesicles. Free fatty acid stock solutions were prepared by dissolving solid oleic acid and stearic acid in analytical-grade 100% methanol and adjusting the concentration to 4.8 mM. The respective stock solutions were stored at −20 °C until further use.

### Quantitative Binding Analyses for Protein–Lipid Interactions by Tryptophan Fluorescence Quenching

The detailed steps of the experimental procedure for determining the binding parameters for PKN1-(HR1) or PKN1-(C2) with lipids are described in Figure S4.^24,30^ The lipid-induced protein tryptophan fluorescence quenching *F*/*F*_o_ value can be defined as quenching % = *F*/*F*_o_ × 100 (%), where *F* is the intrinsic tryptophan fluorescence intensity upon addition of lipid and *F*_o_ is the intrinsic tryptophan fluorescence intensity before lipid addition. The binding curves (equilibrium type) for the recombinant PKN1-(C2) and PKN1-(HR1) proteins with lipids were further analyzed using a nonlinear regression program (SigmaPlot 4.0, USA), whereby two linear segments were drawn based on the best-fit curve to determine the lipid–protein binding molar ratio,^31^ according to *r*/*C*_s_ = p*K*_a_ – *rK*_a_, where *C*_s_ denotes the free lipid concentration, *r* represents the binding function (i.e., lipid-to-protein molar ratio), and *K*_a_ is defined as the association constant.^32^ The lipid–protein saturation values^31^ were determined by a double reciprocal graphic analysis (*F*/*F*_o_)^−1^ against an (infinite total lipid concentration)^−1^. The fractional saturation and *K*_d_ (*K*_a_^–1^) values were calculated by Scatchard analysis, as described previously.^31,32^

### Matrix-Assisted Laser Desorption Ionization-Time-of-Flight Mass Spectroscopy

PKN1-(C2) samples were premixed with the matrix solution consisting of 10 mg/mL sinapinic acid (α-cyano-4-hydroxycinnamic acid was used as the matrix for cardiolipin preparations) in 70% acetonitrile and 0.05% (v/v) aqueous trifluoroacetic acid (TFA) solution. Aliquots (0.5 to 1 μL) of the matrix solutions were applied to the matrix-assisted laser desorption ionization time of flight (MALDI-TOF) target sample disks, followed by 0.5 to 1 μL of aliquots of the recombinant protein or their Lys-C digests. The sample–matrix mixtures were then lyophilized. The instrument was operated in the positive ion-linear mode with a mass accuracy error of <0.5% in the mass range of the polypeptides to be investigated. Mass values were obtained in the linear mode, with calibration based on ribonuclease A (13.7 kDa). The accelerating voltage used was in the range of 20–25 kV and a delayed extraction condition of 600–750 ns was applied to the sample analysis, with the resulting spectra representing the average of 100 laser shots.

### Immunoblotting Analysis

The purified recombinant PKN1 proteins were separated by 10–14% SDS-PAGE and then transferred to nitrocellulose membranes for immunostaining. The membrane was probed with either polyclonal anti-PKN1-(539–556) or rabbit monoclonal anti-PKN1 (Abcam) antibodies at room temperature, before being washed with 1 × Tris buffer saline-Tween-20 (1× TBS-T) wash buffer, followed by addition of secondary antibody (horseradish peroxidase-conjugated goat antirabbit; Silenus or Abcam). The immunoreactive bands were detected by enhanced chemiluminescence (ECL) (Bio-Rad Western ECL substrate or Kodak BioMax).

## Results

### Purification and Characterization of PKN1-(C2), PKN1-(310–473), and PKN1-(25–485)

**Figure 2:**
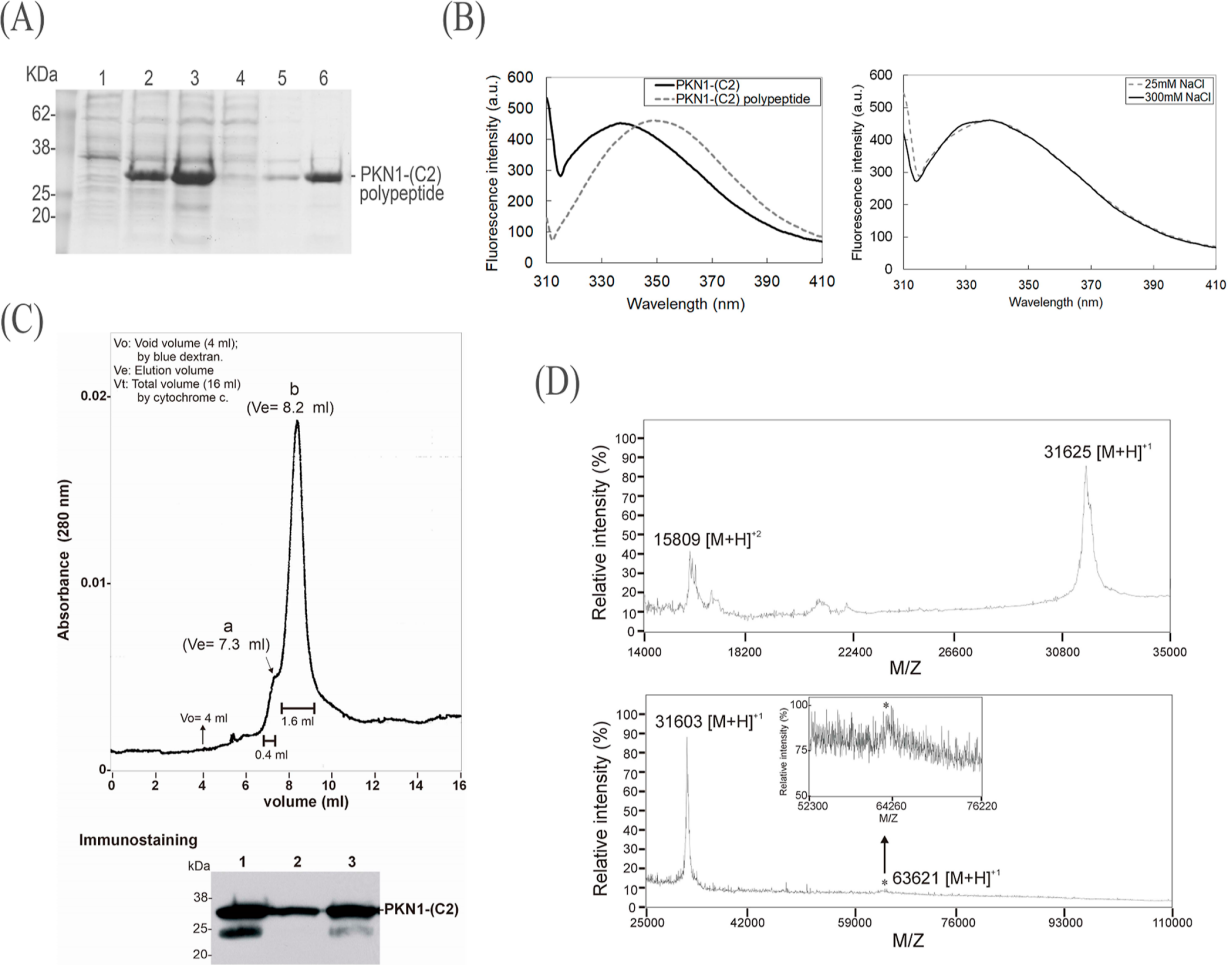
Expression and purification of the PKN1-(C2) protein. (A) PKN1-(C2) was bacterially expressed and recovered from inclusion bodies before being solubilized and affinity-purified in the presence of 8 M urea. Lane 1: cell extracts before induction. Lane 2: cell extracts after induction. Lane 3: induced cell extracts in 8 M urea. Lane 4: the breakthrough fraction. Lane 5: the wash fraction. Lane 6: the affinity-purified C2 polypeptide. (B) Left: Refolding of the PKN1-(C2) polypeptide (1.5 μM) was monitored by intrinsic tryptophan fluorescence spectrometry. The λ_max_ values of the denatured polypeptide (dashed gray line) and refolded protein (solid black line) are 348.5 and 336.6 nm, respectively. Right: Normalized emission fluorescence spectra of PKN1-(C2) in phosphate buffer pH7.4 containing 25 mM (dashed gray line) or 300 mM (solid black line) NaCl at 25 °C. (C) Refolded PKN1-(C2) predominantly eluted in a trimeric conformation under peak “b” in S-200 size-exclusion chromatography (Ve 8.2 mL, 107 kDa), together with a minor shoulder peak “a” (Ve 7.3 mL, 138 kDa) (Table S1). The identity of PKN1-(C2) was confirmed by anti-PKN1-(539–556) immunostaining. (D) Upper: Two molecular ions were detected with apparent masses of 31,625 [M + H]^+^ and 15,809 [M + H]^2+^. Lower: In addition to a 31,603 Da peak, a trace of a 63,621 Da (inset) species was detected.

Recombinant PKN1-(HR1) was successfully purified in a previous study for structural and functional analyses.^24^ For the current study, histidine-tagged PKN1 (residues 342–605) was prepared according to a similar purification strategy as described previously^21^ (Figure 2A; Table S1).

PKN1-(342–605) is hereafter referred to as PKN1-(C2). PKN1-(C2) was identified in the insoluble fraction from *E. coli* cells and affinity-purified under denaturing conditions as polypeptides (Figures 2A and S3). PKN1-(C2) harbors Trp^345^, Trp^410^, Trp^418^, Trp^438^, and Trp^518^ residues, each of which could serve as a conformational probe to monitor the maximal absorption (λ_max_) value for the refolded protein (Figure 1B, left). We determined that a 12 nm blue shift in λ_max_ (349 to 337 nm) was associated with refolding (Figure 2B, left). The λ_max_ value for the tryptophan residues of denatured PKN1-(C2) (348.5 nm) was close to that of free tryptophan (350 nm), indicating that PKN1-(C2) underwent a conformational change during the refolding step, reflecting relocation of tryptophan residues into a more hydrophobic environment. To further characterize the refolded peptide, we compared PKN1-(C2) in the presence of either 25 or 300 mM NaCl and found no detectable difference in its conformation. Next, we subjected PKN1-(C2) to size-exclusion chromatography (Figure 2C). The majority of PKN1-(C2) eluted in peak “b” as a trimer (107 kDa), with a relatively minor shoulder (peak “a”) eluting on the leading edge of the main peak as a tetramer (138 kDa; top panel in Figure 2C). Immunostaining of the collected fractions using anti-PKN1-(539–556) antibody (Figure 1A) confirmed the identity of PKN1-(C2) in both peak fractions (lanes 2–3 in Figure 2C), with some degraded 25 kDa fragment(s) being detectable only in the trimeric fraction (peak b shown in lane 3 in Figure 2C).

Next, we determined the molecular mass of the purified PKN1-(C2) by means of MALDI-TOF mass spectrometry, revealing two peaks, [M + H]^+1^ and [M + H]^+2^, with apparent mass-to-charge ratios (*m*/*z*) of 31,625 and 15*,*809 Da, respectively (Figure 2D). This experimentally determined molecular mass for the 31,625 Da [M + H]^+1^ ion species is consistent, within experimental error, of a predicted mass for PKN1-(C2) of 31,555.1 Da (Figure 1B). We also detected a minor peak in the higher *m*/*z* range of 62–63 kDa (inset, Figure 2D), implying the presence of dimers. The trimeric PKN1-(C2) peak was absent in the presence of the matrix used for MALDI-TOF mass spectrometry (Materials and Methods) where noncovalent interactions among individual subunits might have been disrupted.

In order to address how the PKN1-(HR1) and -(C2) subdomains interact at the molecular level, we also generated histidine-tagged PKN1 (residues 310–473 and 25–485) (Figure 2A; Table S1). PKN1-(310–473) is an AlphaFold program-defined C2 region, and PKN1-(25–485) comprises both HR1 and the above-mentioned C2 region (Figure 1A). PKN1-(310–473) was misfolded and associated with the inclusion bodies where it was solubilized by the presence of 8 M urea and affinity-purified under denaturing conditions as polypeptides (Figures 2A and S2). Unlike PKN1-(C2), the refolding of PKN1-(310–473) polypeptide was unsuccessful and precipitated under experimental conditions similar to those of PKN1-(C2) (see Materials and Methods). Interestingly, PKN1-(25–485) was bacterially expressed in the soluble form that was affinity-purified and confirmed to be in a trimeric conformation by size-exclusion chromatography under native conditions (see Materials and Methods and Figure S3). Importantly, this observation is consistent with the trimeric conformation determined for PKN1-(C2)-(342–605) (Figures 1 and 2).

### Interactions between PKN1-(C2) and Phospholipids

**Figure 3:**
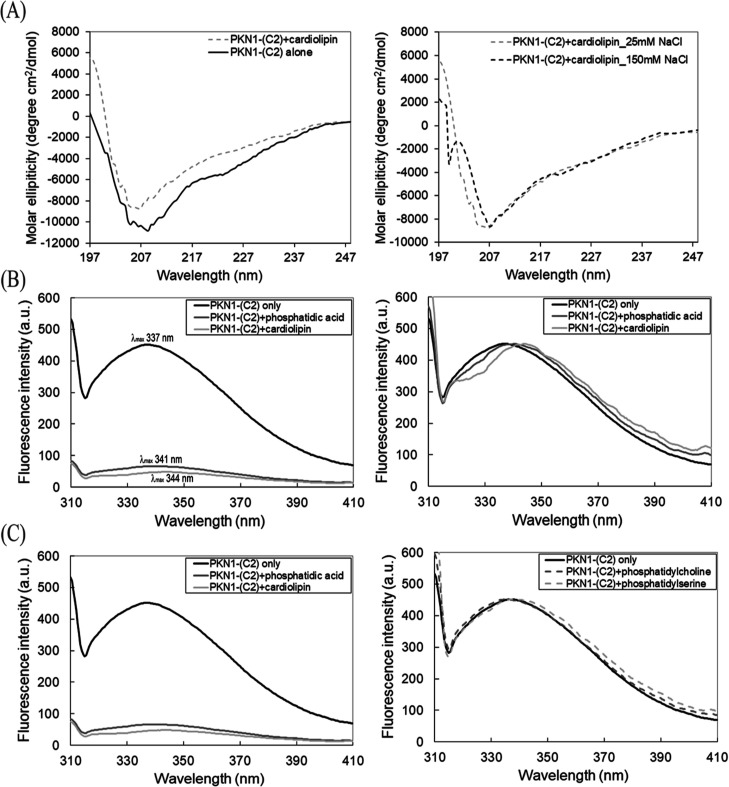
Structural impacts of phospholipid binding to PKN1-(C2). (A) Left: PKN1-(C2) (1.7 μM) was analyzed by CD spectroscopy in 20 mM phosphate buffer pH 7.4 and 25 mM NaCl at 25 °C. The spectra were recorded in the absence (solid black line) or presence (dashed gray line) of 6.6 μM cardiolipin. Right: The CD spectra of premixed PKN1-(C2) (1.7 μM) and cardiolipin (6.6 μM) in the presence of 25 mM (dashed gray line) or 150 mM (dashed black line) NaCl. (B,C) Left: The original intrinsic tryptophan fluorescence spectra of 0.4 μM PKN1-(C2) in the absence (solid black line) or presence of cardiolipin (solid pale gray line), phosphatidic acid (solid dark gray line), phosphatidylcholine (dashed dark gray line), or phosphatidylserine (dashed pale gray line). Right: the fluorescence intensity of the respective protein–lipid binding signal was normalized to the λ_max_ value of the PKN1-(C2) only in fluorescence emission spectra. A protein/lipid molar ratio of 1:10 was used in all experiments.

Our identification of five putative amphipathic cardiolipin-binding motifs located within the PKN1-(C2) sequence raises the possibility that PKN1-(C2) could exert phospholipid-binding functions similar to those of the PKN1-(HR1) domain (Figure 1B). We investigated lipid–protein interactions by means of CD or a tryptophan fluorescence-quenching assay (Figure S6), as deployed previously to establish the lipid-binding constant for PKN1-(HR1).^24^ The CD spectrum of PKN1-(C2) in the absence of lipid revealed that in solution, it consists of 12.3% α helix and 51.9% β sheet (Figure 3A).

Addition of 6.6 μM cardiolipin altered the spectrum, giving rise to estimates of 9.5% α helix and 65.1% β sheet for the PKN1-(C2) CD signal at a wavelength of 205–210 nm (Figure 3A, left). This outcome is distinct to the signal decrease detectable for the cardiolipin and PKN1-(HR1) interaction in our previous CD analysis.^24^ Therefore, we postulate that binding of cardiolipin increased by up to 10% in the secondary structural conformation of PKN1-(C2). Moreover, we detected no significant difference in the CD spectra of PKN1-(C2) in the presence of cardiolipin (1:4 molar ratio of protein to lipid) when the concentration of NaCl was increased from 25 to 150 mM (Figure 3A, right).

To further characterize binding between PKN1-(C2) and cardiolipin as well as other phospholipids that may modulate the enzymatic activity of PKN1, we employed tryptophan intrinsic fluorescence spectrometry. Our results show that cardiolipin, phosphatidic acid, phosphatidylserine, and phosphatidylcholine quenched the Trp fluorescence emission signal of PKN1-(C2) at 340 nm by 90, 86, 82, and 75%, respectively, supporting that the five Trp residues located within the C2-like domain of PKN1 could effectively function as indicators of binding between the target protein and these phospholipids (Figure 3B,C). Furthermore, in the absence of phospholipid, the λ_max_ value determined for PKN1-(C2) was 337 nm, which was red-shifted to 341 and 344 nm in the presence of phosphatidic acid and cardiolipin, respectively, in the normalized results (see the normalized fitted profiles in the right panel of Figure 3B). In contrast, no detectable variation in λ_max_ was found for either phosphatidylserine or phosphatidylcholine (Figure 3C, right panel). Thus, the binding of cardiolipin or phosphatidic acid seems to exert relatively stronger effects on the conformation of PKN1-(C2) than the other two tested phospholipids.

**Figure 4:**
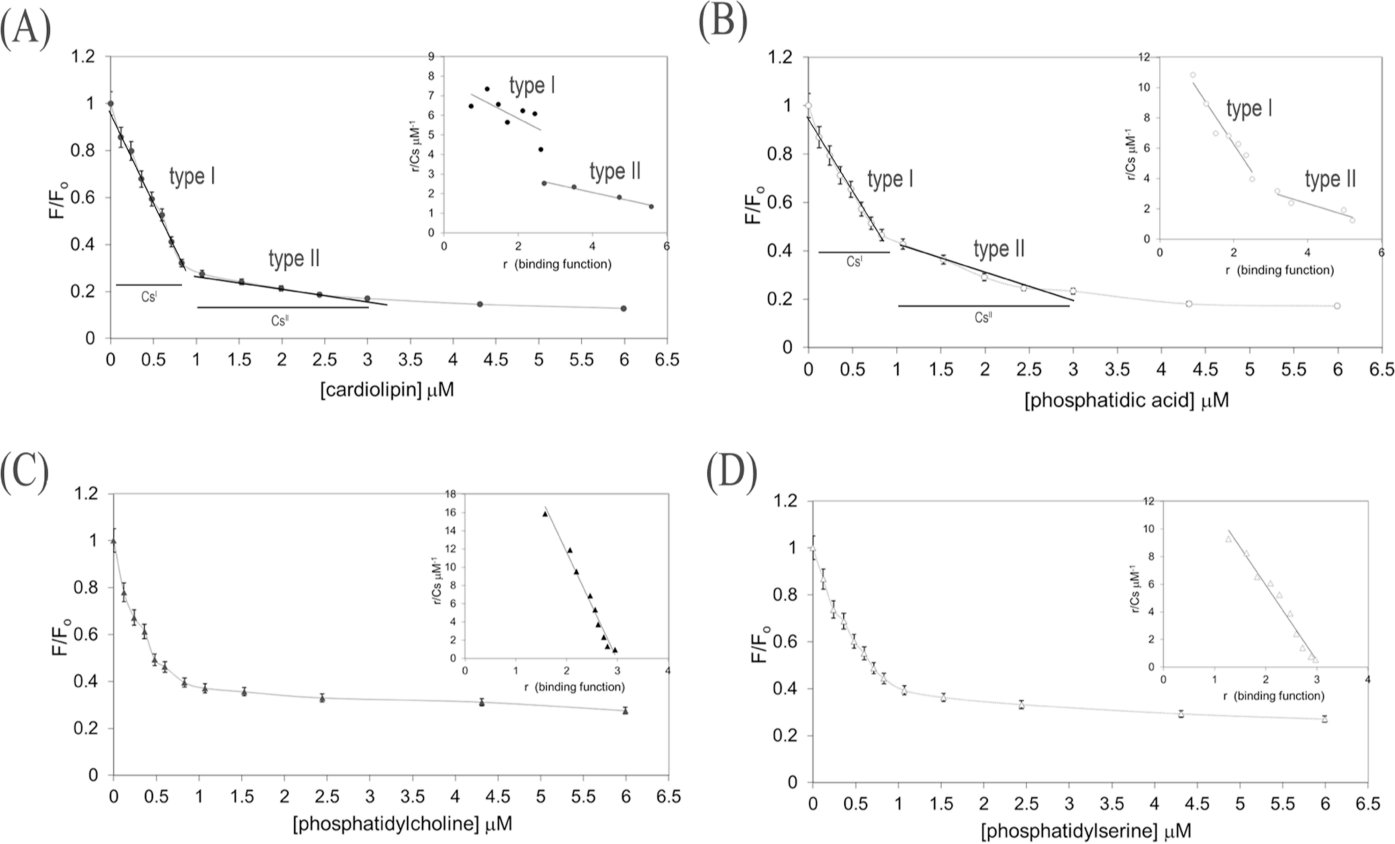
Distinct phospholipid-binding characteristics of PKN1-(C2). Interactions between PKN1-(C2) and phospholipids were measured as a function of the intrinsic tryptophan fluorescence quenching. PKN1-(C2) (0.3 μM) was titrated with phospholipids in a buffer containing 20 mM phosphate buffer, pH7 4, and 25 mM NaCl at 25 °C. The protein–phospholipid binding curves are marked by closed circles for cardiolipin (A), open circles for phosphatidic acid (B), closed triangles for phosphatidylcholine (C), and open triangles for phosphatidylserine (D). In (A) and (B), the bars in the graph marked as Cs^I^ and Cs^II^ define the range of free lipid concentrations for either type I or II interactions with PKN1-(C2). Lipid-to-protein stoichiometries were measured empirically by extrapolation with linear segments from their respective titration curves, which in both cases yielded a lipid-to-protein binding molar ratio.^24^ Scatchard plot analyses (inset) were conducted on the binding data, and the binding parameters are summarized in Table 1.

To further assess the binding affinity of PKN1-(C2) for phospholipids, we again deployed tryptophan fluorescence emission spectrometry to determine their binding constants. The PKN1-(C2) binding curves for both cardiolipin and phosphatidic acid appeared to be biphasic, with Scatchard plots (insets, Figure 4A,B) indicating the presence of high- and low-affinity sites, referred to as “type I” and “type II” interactions, respectively (see the model discussed in Figure S5).

The intercept value(s) obtained from *r* (binding function) in the Scatchard plots was estimated to reflect three “type I” and six “type II” sites within PKN1-(C2) (Figure 4A,B). Dissociation constants (*K*_d_) were then calculated, taking into account phospholipid-binding stoichiometries in the range of 0.1–2.4 μM (summarized in Table 1), together with the data on binding of phosphatidylcholine and phosphatidylserine to PKN1-(C2) shown in Figure 4C,D, respectively. The linear Scatchard plots (insets, Figure 4C,D) and the abscissa intercepts reflect three phospholipid-binding sites in PKN1-(C2) (Figure 4C,D; Table 1).

**Table 1:** Binding Parameters for PKN1-(C2) with Phospholipids

phospholipid	*K*_d_^c^	lipid/protein molar ratio^d^
cardiolipin^a^	1.0 ± 0.6 μM	3 (type I)
cardiolipin^a^	2.4 ± 0.2 μM	6 (type II)
phosphatidic acid^a^	0.3 ± 0.0 μM	3 (type I)
phosphatidic acid^a^	1.3 ± 0.4 μM	6 (type II)
phosphatidylcholine^b^	0.1 ± 0.0 μM	3
phosphatidylserine^b^	0.2 ± 0.0 μM	3

a Data obtained from Figure 4A,B.

b Data obtained from Figure 4C,D.

c *K*_d_ values were calculated as described in Materials and Methods.

d The calculations are based on the assumption that PKN1-(C2) was present predominantly as a trimer (Figure S5).

### Influence of Ionic Strength on Interactions of PKN1-(C2) with Phospholipids

**Figure 5:**
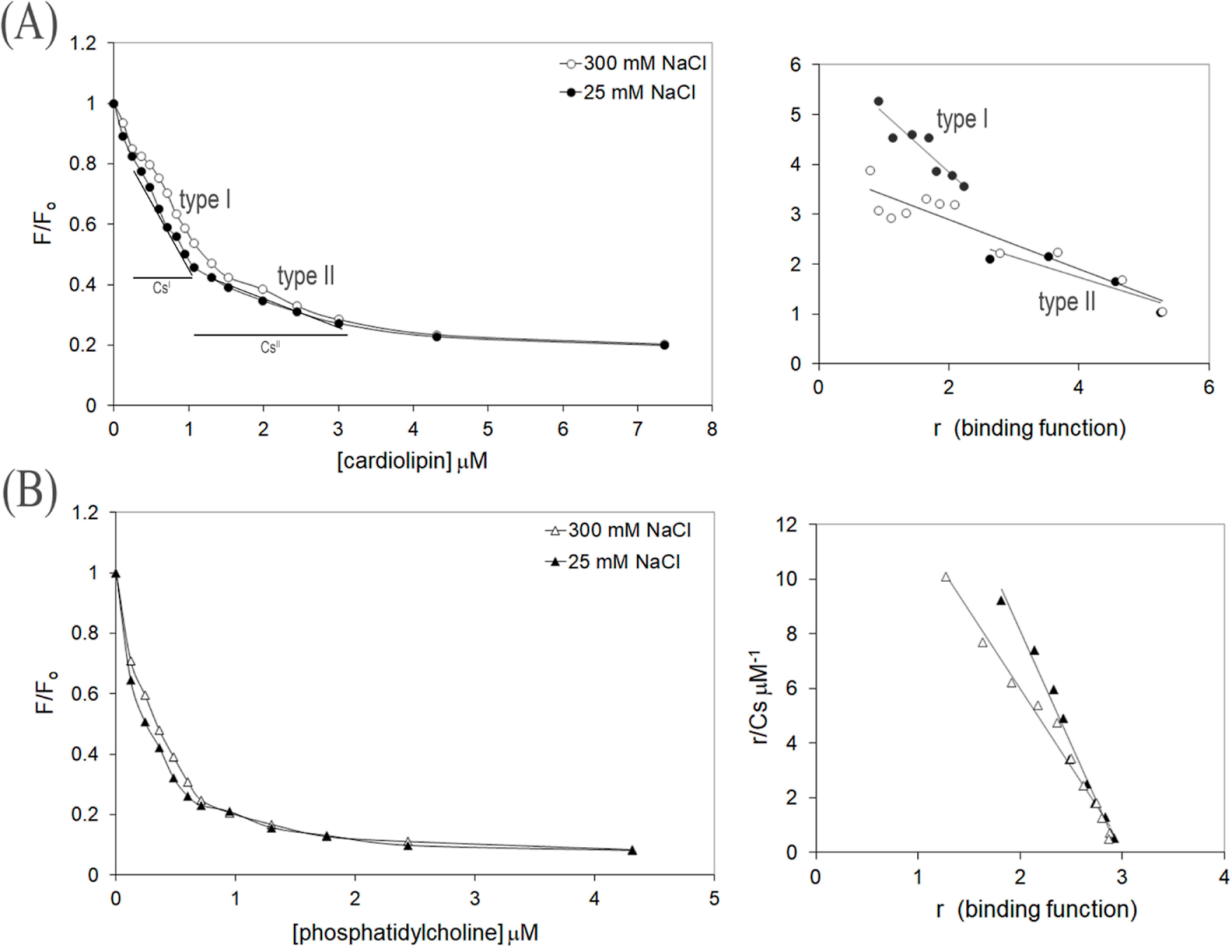
Influence of ionic strength on the binding curves for PKN1-(C2) with either cardiolipin or phosphatidylcholine. Left: the interactions between PKN1-(C2) and (A) cardiolipin or (B) phosphatidylcholine were measured as a function of intrinsic tryptophan quenching fluorescence. PKN1-(C2) at 0.4 or 0.17 μM was titrated with cardiolipin and phosphatidylcholine in a buffer containing 20 mM phosphate buffer pH 7.4 with either 25 mM NaCl (closed symbols) or 300 mM NaCl (open symbols) at 25 °C. Right: Scatchard plot analyses were conducted on the binding data, and the binding parameters are summarized in Table 2.

Our identification of two types of binding sites for cardiolipin or phosphatidic acid in PKN1-(C2) is intriguing (Figure 4A,B). A previous study revealed that both polar and nonpolar components are involved in interactions between PKN1-(HR1) and cardiolipin or phosphatidic acid.^24^ In order to further characterize binding between PKN1-(C2) and phospholipids, we conducted protein–lipid binding experiments at NaCl concentrations of 25 and 300 mM. Notably, the high-affinity type I interaction between PKN1-(C2) and cardiolipin only occurred at 25 mM NaCl (*K*_d_ = 0.8–1.0 μM), since it was abolished in the presence of 300 mM NaCl (Figure 5A; Table 2).

**Table 2:** Effects of Ionic Strength on Binding Parameters of PKN1-(C2)^d^ with Either Cardiolipin or Phosphatidylcholine

			lipid/protein molar ratio lipid/protein	
phospholipids	*K*_d_^25^^c^^,^^e^	*K*_d_^300^^c^	25 mM	300 mM
cardiolipin site I binding	0.8 ± 0.2^a^μM	-d	3	
cardiolipin site II binding	2.4 ± 0.9^a^μM	2.0 ± 0.3^a^μM	6	6
phosphatidylcholine binding	0.1 ± 0.0^b^μM	0.2 ± 0.0^b^μM	3	3

a Data obtained from Figure 5A.

b Data obtained from Figure 5B.

c *K*_d_ values were calculated as described in Materials and Methods.

d No evidence of type I binding.

e Standard errors of the *K*_d_ determinations were generated from the linear regression analyses of Scatchard plot data.

In contrast, the low-affinity type II PKN1-(C2) and cardiolipin interaction was relatively unaffected upon increasing the salt concentration, with *K*_d_ values for 25 and 300 mM NaCl of 2.4 and 2.0 μM, respectively (Figure 5A; Table 2), implying a predominantly hydrophobic type of protein versus lipid interaction(s). Interestingly, phosphatidylcholine bound to PKN1-(C2) with the highest affinity among all of the lipids we tested, with that interaction proving insensitive to a high salt concentration (Tables 1 and 2). Thus, phosphatidylcholine exhibits high-affinity binding to PKN1-(C2) but, unlike high-affinity cardiolipin binding, this interaction is predominantly hydrophobic (Figure 5B and Table 2).

### Binding Constants for PKN1-(C2) with Fatty Acids

**Figure 6:**
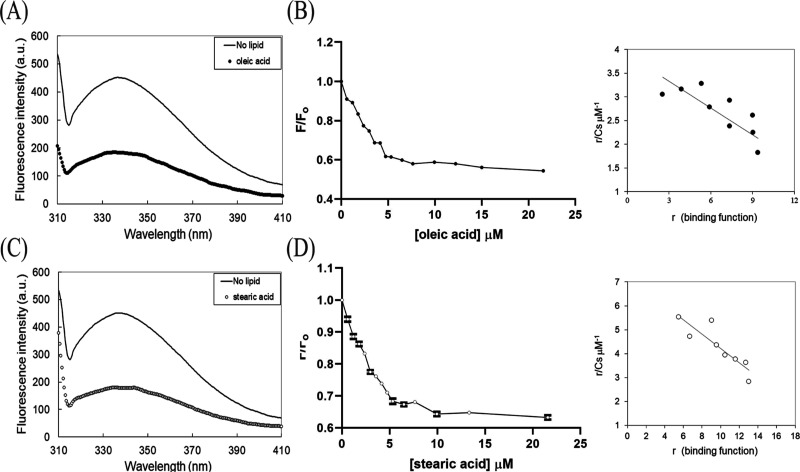
Interactions between PKN1-(C2) and C18 fatty acids. Emission fluorescence spectra of 0.3 μM PKN1-(C2) in the absence or presence of (A) oleic acid (closed circles) or (C) stearic acid (open circles) in 20 mM phosphate buffer pH 7.4 and 25 mM NaCl at 25 °C. The molar ratio of fatty acid to protein was 20. The binding curve for PKN1-(C2) with either (B) oleic acid or (D) stearic acid was measured as a function of the intrinsic tryptophan quenching fluorescence. Scatchard plots (right panel), derived from the binding curves shown in (A) or (C), are based on estimated stoichiometry, with the line of best fit for the binding data shown. The binding parameters are summarized in Table 3.

In addition to phospholipids, previous studies have established that unsaturated C18–C20 fatty acids are potent activators of both native and recombinant forms of PKN1.^10,27^ In particular, it has been demonstrated that oleic acid elicited a 3-fold increase in kinetic efficiency of substrate phosphorylation by PKN1 relative to arachidonic acid.^10^ Hence, we focused on determining the interactions between PKN1-(C2) and C18 oleic (unsaturated) and stearic (saturated) acids. To assess fatty acid and recombinant protein interactions, we determined the intrinsic tryptophan fluorescence spectra of PKN1-(C2) in the absence and presence of either oleic or stearic acid. We observed that both of these fatty acids induced 59% fluorescence quenching of PKN1-(C2) at a wavelength of λ_340nm_ (Figure 6A–C).

PKN1-(C2) was titrated by either oleic or stearic acid as a function of lipid concentration, giving rise to 12 and 17 molecules of oleic and stearic acids bound, respectively, to a trimeric PKN1-(C2) (estimated from the intercept value for *r* in the Scatchard plot of Figure 6B–D). We also determined *K*_d_ values of 5.3 and 3.3 μM, respectively, for binding of oleic and stearic acids to PKN1-(C2), which are not significantly different (*p* > 0.05) (Table 3).

**Table 3:** Binding Parameters for PKN1-(C2) and Fatty Acid Interactions

fatty acid	*K*_d_^c^^‡^	stoichiometry of lipid/protein
oleic acid^a^	5.3 ± 1.4 μM^d^	12
stearic acid^b^	3.3 ± 0.8 μM^d^	17

a Data obtained from Figure 6B.

b Data obtained from Figure 6D.

c *K*_d_ values were calculated as described in Materials and Methods.

d Not statistically significant (*p* value > 0.05).

## Discussion

In this study, we have identified cardiolipin-binding sequences in PKN1-(C2) that are similar to the amphipathic motifs clustered in PKN1-(HR1)^24^ (Figure 1B). Furthermore, our CD spectroscopic analysis has revealed that the majority of PKN1-(C2) folds into a β-strand-enriched conformation (Figure 3A). In contrast to the disruptive effects observed for binding of cardiolipin to PKN1-(HR1),^24^ cardiolipin induced significant conformational changes to PKN1-(C2) by increasing its secondary structural contents. Interestingly, sequence alignment demonstrates that the primary structures of PKN1-C2-(398–471) and nPKCε-C2-(44–123) are highly homologous (Figure S1A), consistent with the three-dimensional structure of the nPKC C2-like domain (Figure S1B).^26^ Our binding analyses indicate that PKN1-(C2) is a target site for phospholipid and free fatty acid interactions. For instance, binding of the PKN1-activating phospholipids cardiolipin or phosphatidic acid-induced detectable conformational changes in PKN1-(C2), as revealed by both CD spectroscopy and intrinsic fluorescence spectrometry (Figure 3). Intriguingly, binding of PKN1-(C2) to either cardiolipin or phosphatidic acid appears to be biphasic (Figure 4), which differs from binding of PKN1-(HR1) to cardiolipin, as revealed by a previous study.^24^ For instance, increased salt levels (from 25 to 300 mM) reduced the binding affinity of PKN1-(HR1) for phospholipid 5-fold, i.e., to a level comparable to that for C18 fatty acids (*K*_d_ = 140–200 nM) (Figure S6 and Table S3), reflecting the typically amphipathic nature of binding between PKN1 and phospholipids. In contrast, the type I binding between PKN1-(C2) and cardiolipin was abolished under the high salt condition, whereas the type II binding remained relatively unaffected, implying a distinct binding pattern to that of PKN1-(HR1). The amphipathic L(I)R(K)X motifs identified within the C2-like region may represent a limited number of binding interfaces between PKN1 and phospholipids (Figure 1B). In addition, only expression of both HR1 and the C2 region [i.e., PKN1-(25–485)] renders the C2 region [i.e., PKN1-(310–473)] soluble under native conditions as a trimer. Therefore, we suggest that folding of the functional C2 region is dependent on the presence of PKN1-(HR1) (Figures S2 and S3). Hence, the molecular mechanism of PKN1 versus phospholipid interactions might involve both HR1 and C-like domains. For example, binding of cardiolipin mildly reduced the α-helical nature of PKN1-(HR1),^24^ but it significantly increased the β strand content of its C2-like counterpart (Figure 3A), with this latter potentially reducing the free energy required for kinase activation. In contrast, non-PKN1-activating phosphatidylcholine and phosphatidylserine only appear to bind to high-affinity (type 1) site(s). Nevertheless, the *K*_d_ value (0.1–0.2 μM) determined for PKN1-(C2) in the current study is consistent with binding constants previously determined for full-length PKCε(*K*_d_, 0.1–0.4 μM).^9^ Therefore, we speculate that the C2-like domain of PKN1 plays a functional role in lipid binding, possibly relating to the type of vesicle bilayer association, i.e., in a manner comparable to that of nPKCs. The insensitivity of phosphatidylcholine and low-affinity cardiolipin-binding events to our high salt conditions (300 mM NaCl) indicates that hydrophobic interactions between the phospholipid acyl side chains and hydrophobic-rich regions of PKN1-(C2) are important determinants of these binding events (Figure 1B).

Although our results demonstrate that both cardiolipin and phosphatidylcholine bind to the hydrophobic site(s) of PKN1-(C2), they do not reveal if the two phospholipids share common hydrophobic binding site(s). In fact, the differences we observed in their binding affinities with PKN1-(C2) and respective stoichiometries instead indicate distinct binding sites for these two types of phospholipids (Figures 4 and 5; Tables 1 and 2). Another difference in the binding characteristics of cardiolipin and phosphatidylcholine with PKN1-(C2) is that only high-affinity cardiolipin binding (type I) was inhibited by a high salt concentration (300 mM) (Figure 5; Table 2). This property implies that electrostatic binding components are more likely to be disrupted and, consequently, less likely to be involved in physiological contexts. The fact that we detected no marked variation in CD spectroscopy for PKN1-(C2) in the presence of cardiolipin upon increasing NaCl from 25 to 150 mM indicates that hydrophobic (type II) components (i.e., fatty acids) seem to play a more important role in binding between the peptide and the PKN1-activating lipid (Figure 3A). A previous site-directed mutagenesis study on the C2-like domain of nPKCε confirmed that hydrophobic residues play more significant roles than electrostatic components in binding to phospholipids.^26^ Despite our study revealing that PKN1-(C2) has similar binding affinities for oleic acid and stearic acid, previous study has shown that the former promotes by 2- to 3-fold the kinetic efficiency of PKN1’s substrate phosphorylation relative to the latter.^10^ In addition, our previous study indicated that one molecule of tested phospholipid was bound to each molecule of PKN1-(HR1).^24^ Accordingly, it was estimated that at least 2 to 4 of the acyl side chains were involved in binding to one PKN1-(HR1) molecule. Herein, consistently, we determined the C18 fatty acid-to-protein molar ratio to be 3 or 4 (Table S3). Furthermore, we also found that phospholipid bound to trimeric PKN1-(C2) at molar ratios of 3 to 6 (Tables 1 and 2). In the case of cardiolipin, the number of acyl side chains estimated to interact with PKN-(C2) ranged between 12 and 24. Indeed, the C18 fatty acid-to-protein molar ratio was determined to be 12 or 17 (Table 3). The relatively lower binding affinity of PKN1-(C2) for C18 fatty acids relative to cardiolipin is consistent with kinetic data from a previous study.^10^ For instance, the *K*_d_ value we determined for binding of PKN1-(C2) to C18 fatty acid is 3–5 μM (Table 3), consistent with an EC_50_ value of 4.2 μM for oleic acid-mediated activation of PKN1.^10^ In addition, the EC_50_ value of 1.7 μM determined previously^10^ for cardiolipin-driven PKN1 activation is consistent with our determined *K*_d_ value of 1–2.4 μM for PKN1-(C2) interaction (Tables 1 and 2). The relevance of the aforementioned protein–lipid binding results to the previously observed kinetic data determined for full-length PKN1 enzyme successfully demonstrates how the C18 acyl composition of the phospholipid is directly involved in modulating PKN1’s activity. Hence, these data support that both PKN1-(HR1) and -(C2) cooperatively play key roles in regulating PKN1 activity.

**Figure 7:**
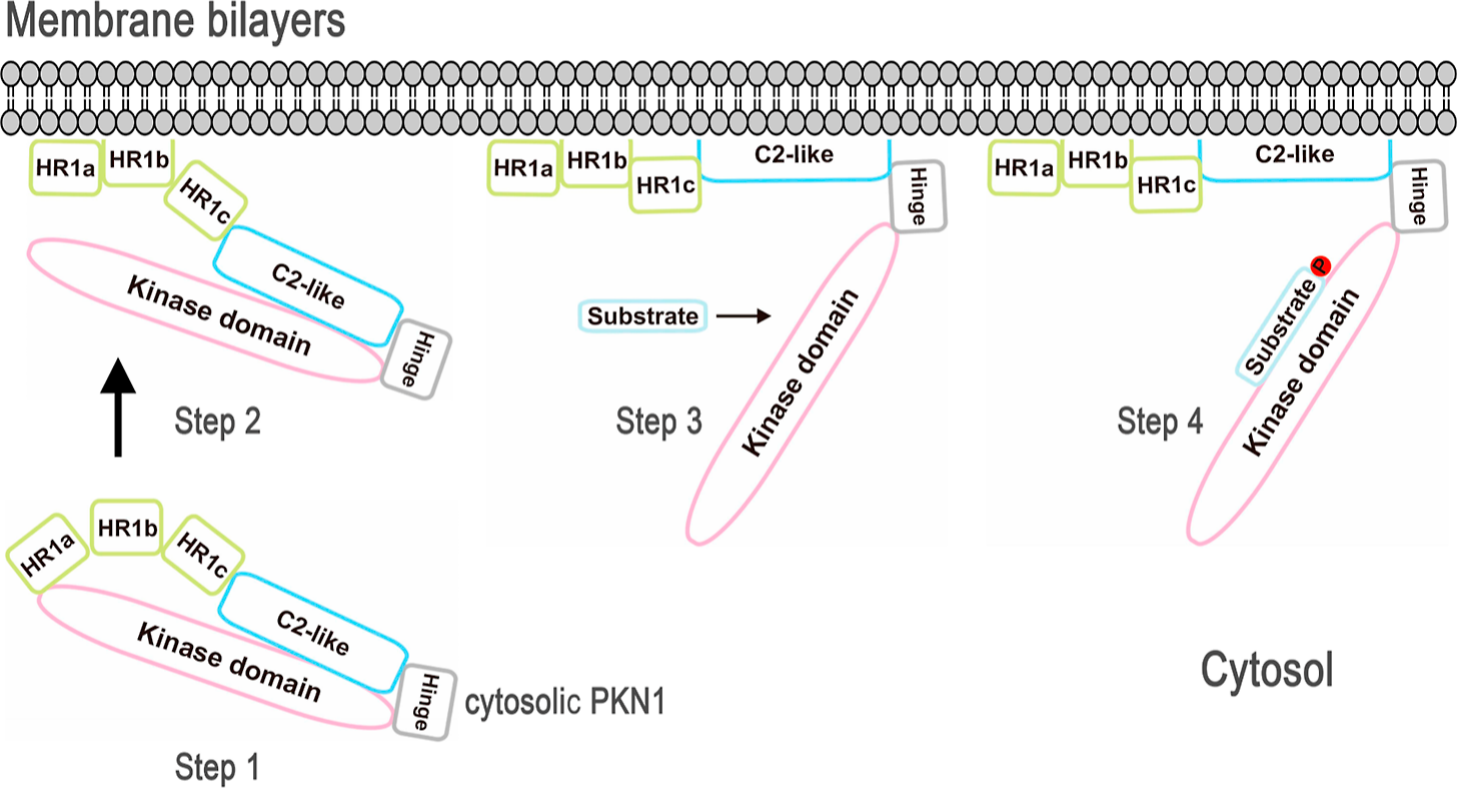
Proposed model for the regulatory and activation steps of PKN1. Proposed simplified model for the HR1- and C2-like-mediated regulatory and activation steps of PKN1. Step 1: the cytosolic PKN1 enzyme is recruited to the membrane via the phosphatidylcholine-binding site on its HR1b subdomain. Step 2: binding of the acidic phospholipid components to the high-affinity amphipathic binding sequence on PKN1-(HR1a) partially relieves the autoinhibitory effect imposed by the HR1 pseudosubstrate motif. Step 3: PKN1-(C2) interacts with the membrane to disrupt the C2-like pseudosubstrate sequence, thereby releasing the catalytic domain to expose the active site. Step 4: the active site of the PKN1 catalytic domain becomes accessible for substrate entry and, consequently, binding and phosphorylation.

The overall lipid-binding affinity for PKN1-(HR1)^24^ is 10-fold greater than that determined for PKN1-(C2) in this study. Notably, a phosphatidylcholine-binding motif conserved within PKN1-(HR1) is absent from PKN1-(C2),^24^ which might have resulted in weaker and nonspecific interactions. We postulate that cytosolic PKN1 remains in an autoinhibited state with the putative phosphatidylcholine-binding motif of HR1b directing PKN1 membrane recruitment (Figure 7).

Membrane-bound PKN1 facilitates high-affinity cardiolipin binding to HR1a via amphipathic contacts with acidic phospholipids to disrupt the HR1a pseudosubstrate motif (Figure 7). PKN1 can then be activated and autophosphorylated.^5,33^ PKN1-(C2) forms hydrophobic interactions with the membrane to displace the C2 pseudosubstrate sequence, thereby releasing the catalytic domain (Figure 7) and enabling substrate binding and phosphorylation (Figure 7). Further investigations are necessary to reveal the structural basis of how PKN1-(C2) interacts with the PKN1 catalytic domain.

## Supporting Information Available

S-200 size-exclusion chromatographic fractions of PKN1-(C2), lipids used in this study, binding parameters for PKN1-(HR1) and fatty acid interactions, sequence alignment of PKN1-C2-(398–471) and nPKCε-C2-(44–123) and the X-ray crystal structure of nPKCFε-C2-(44–123), solubilization of PKN1-(310–473) under denaturing condition and its affinity purification as polypeptide, PKN1-(25–485) protein adopting a trimeric conformation under native conditions, experimental strategy for investigating PKN1 binding interactions with lipids, model describing the PKN1-(C2) interactions with cardiolipin or phosphatidic acid, and binding curves for interactions of PKN1-(HR1) with fatty acids (PDF)

## Accession Codes

UniProtKB Q63433

## Abbreviations

PKCs: protein kinase C
PKN1: PKC-related serine/threonine protein kinase N1
HR: homologous region
MALDI-TOF MS: matrix-assisted laser desorption ionization time-of-flight mass spectrometry
