# Supplementary material for: Lipid-Binding Regions within PKC-Related Serine/Threonine Protein Kinase N1 (PKN1) Required for Its Regulation
Source: Biochemistry. 2024 Mar 5;63(6):743–53. doi: 10.1021/acs.biochem.4c00009 (PMC10956426; doi:10.1021/acs.biochem.4c00009)
Supplement: Supplementary file 1 — bi4c00009_si_001.pdf [file bi4c00009_si_001.pdf]

Supporting Information

Lipid-binding regions within PKC_related serine/threonine_protein kinase N1 (PKN1) required for its regulation

Jason L.J. Lin[1,3]* and Hanna S. Yuan[2]

[1]Genomics Research Center, Academia Sinica, Taipei 11529, Taiwan.

[2]Institute of Molecular Biology, Academia Sinica, Taipei 11529, Taiwan.

[3]Department of Biochemistry and Molecular Biology, University of Melbourne, Victoria 3010 Australia.

This file contains:
Supporting Information Tables S1-S3 and Figures S1-S6

*Address correspondence to:

Jason L.J. Lin

Tel: +886-2-2789-8072

Fax: +886-2-2789-8811

E-mail: jasonlin@gate.sinica.edu.tw

Table S1
S-200 size exclusion chromatographic fractions of PKN1-(C2).

S-200 column fractions	Total fraction size‡	Ve* (ml)	[protein] μg/ml	Total protein (μg)
Refolded PKN1-(C2) (Sample loaded before S-200)	0.44 ml	–	212	93.3
peak a	0.4 ml	7.3	7	2.8
peak b	1.6 ml	8.2	46	73.6
Protein recovery	-	-	-	82%

* Ve: the elution volume of each protein peak.
‡The sizes of peak fractions are as indicated in Fig. 2 (C).

Table S2
Lipids used in this study.

Common name	Chemical name	Source	Acyl fatty acid %*
Cardiolipin[1]	Diphosphatidyl-Glycerol	Bovine heart	87% linoleic acid (C18:2). 8% oleic acid (C18:1). 5% other fatty acid.
Phosphatidic acid[2]	1,2-Diacyl-sn-glycero l-3-phosphate	Fresh egg yolk[2]	34%, palmitic acid (C16:0). 32%, oleic acid (C18:1). 18%, linoleic acid (C18:2). 11%, stearic acid (C18:0). 5% other fatty acid.
Phosphatidylserine[3]	1,2-Diacyl-sn-glycero l-3-phospho-$_L$-serine	Bovine brain	42% stearic acid (C18:0). 34%, oleic acid (C18:1). 8% docosahexaenoic acid (C22:6). 16% other fatty acid.
Phosphatidylcholine[3]	1,2-Diacyl-sn-glycero l-3-phosphocholine	Fresh egg yolk[2]	34%, palmitic acid (C16:0). 32%, oleic acid (C18:1). 18%, linoleic acid (C18:2). 11%, stearic acid (C18:0). 5% other fatty acid.
Oleic acid[4]	*cis*- Octadecenoic acid	Chemically synthesized	–
Stearic acid[4]	Octadecenoic acid	Chemically synthesized	–

1. in ethanol, 2. in sodium salt**,** 3. in lyophilized powder, and 4. free acid.
* Acyl fatty acid compositions (Avanti® Polar Lipids, Inc., 2002-03).

Table S3

Binding parameters for PKN1-(HR1) and fatty acid interactions[#].

Fatty acid	Dissociation constant K_d*	Stoichiometry Lipid / protein (mol/mol)
Oleic acid	139 ± 34 nM[†]	3
Stearic acid	201 ± 82 nM[†]	4

#: The binding parameters were calculated from the data presented in Fig. S6.
*: K_d values were estimated as described in MATERIALS AND METHODS.
†: Not statistically significant (p value > 0.05).

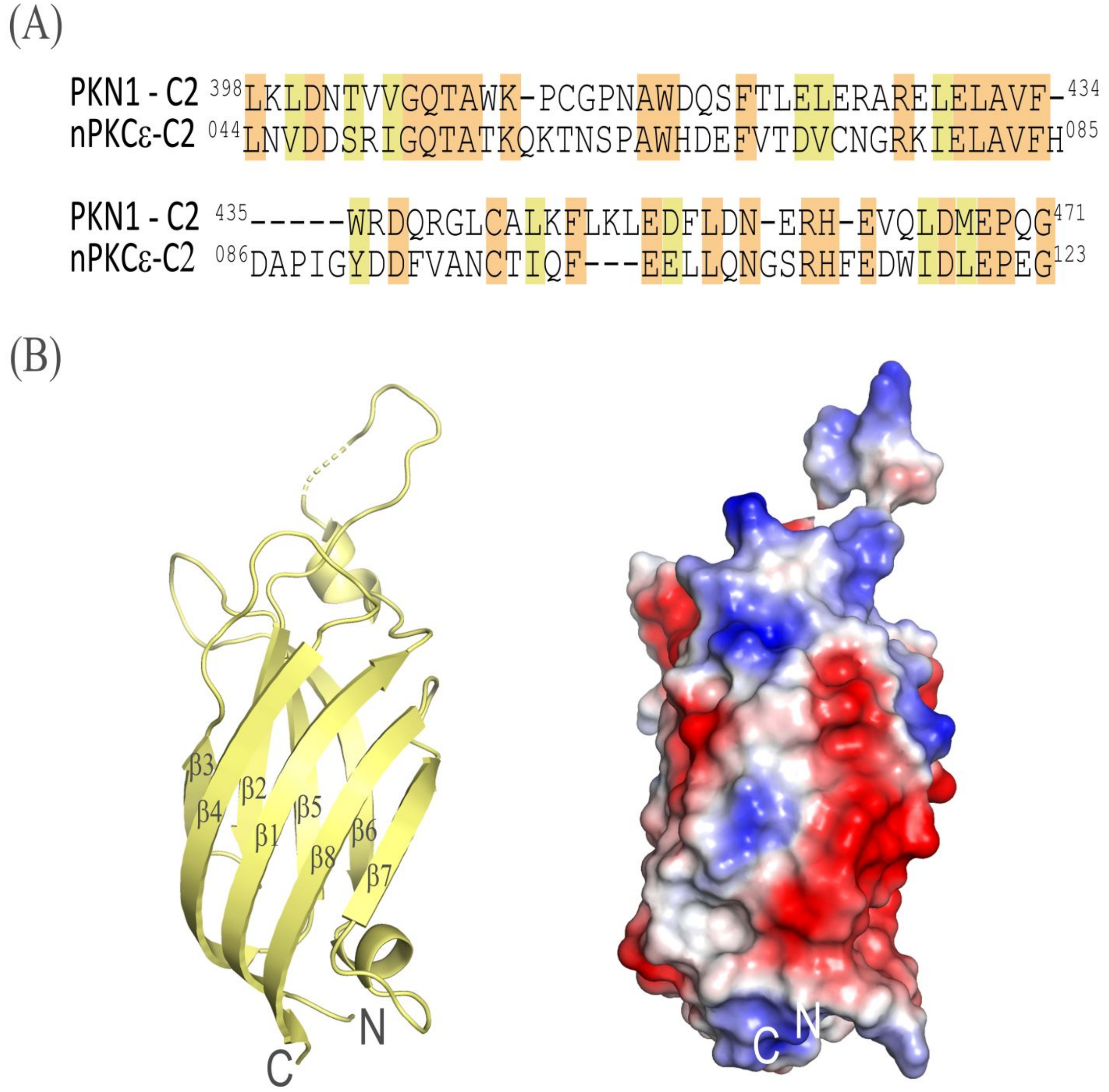

Figure S1. Sequence alignment of PKN1-C2-(398-471) and nPKCε-C2-(44-123), and the X-ray crystal structure of nPKCε-C2-(44-123).

(A) Sequence alignment of PKN1-C2-(398-471) and nPKCε-C2-(44-123), revealing they share up to 53% similarity (highlighted in yellow and orange) and 35% identity (highlighted in orange). (B) Left: the crystal structure of nPKCε-C2-(2-136) (PDB ID: 1GMI) reveals that the majority of the nPKC C2-like domain is folded in an anti-parallel β-stranded conformation. Right: Both electrostatic (negative in red, positive in blue) and hydrophobic (in white) regions are present on the C2-like domain of nPKCε.

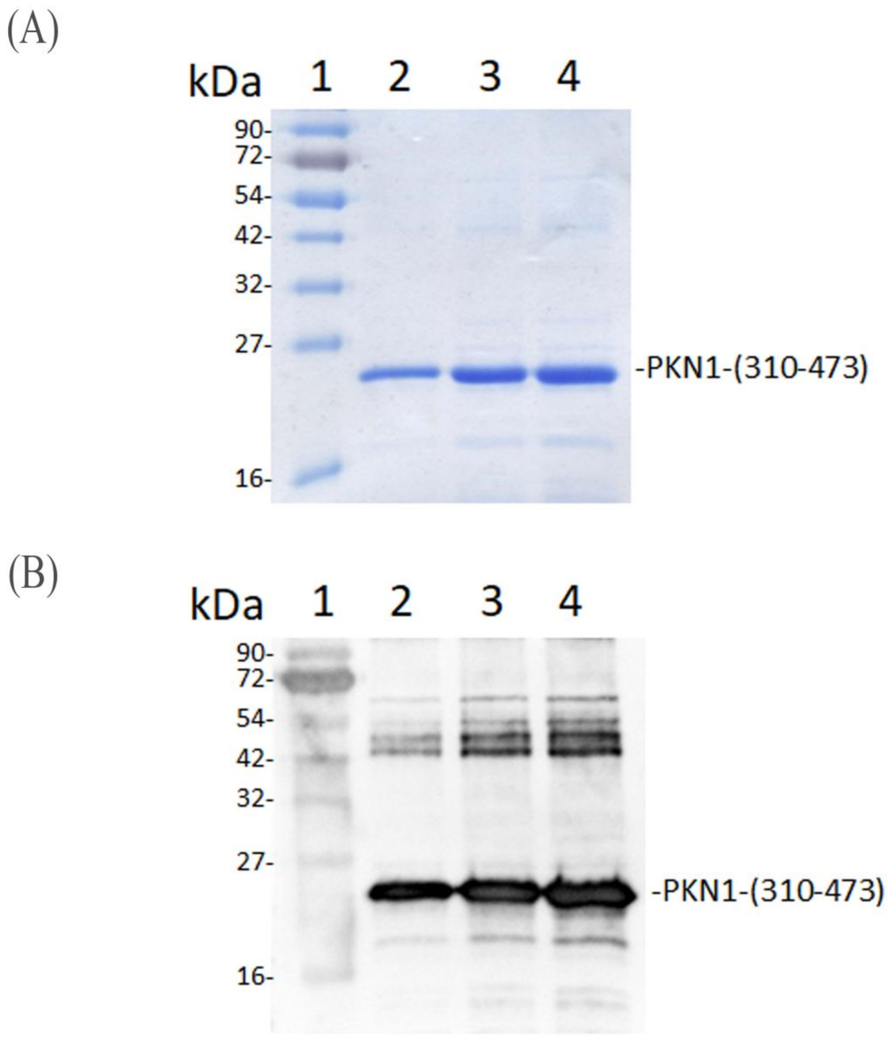

Figure S2. PKN1-(310-473) was solubilized under denaturing condition and affinity purified as polypeptide.

PKN1-(310-473) corresponding to the Alphafold-defined C2 region was purified by metal ion affinity chromatography as a polypeptide in the presence of 8M urea and further analyzed by (A) 14% SDS-PAGE followed by Coomassie blue and (B) immunostaining using rabbit monoclonal anti-PKN1 (Abcam). The estimated molecular weight for PKN1-(310-473) is 20.49 kDa. Lane 1: protein size markers, lane 2: 0.5 μg of PKN1-(310-473), lane 3: 1.0 μg of PKN1-(310-473), and lane 4: 1.5 μg of PKN1-(310-473).

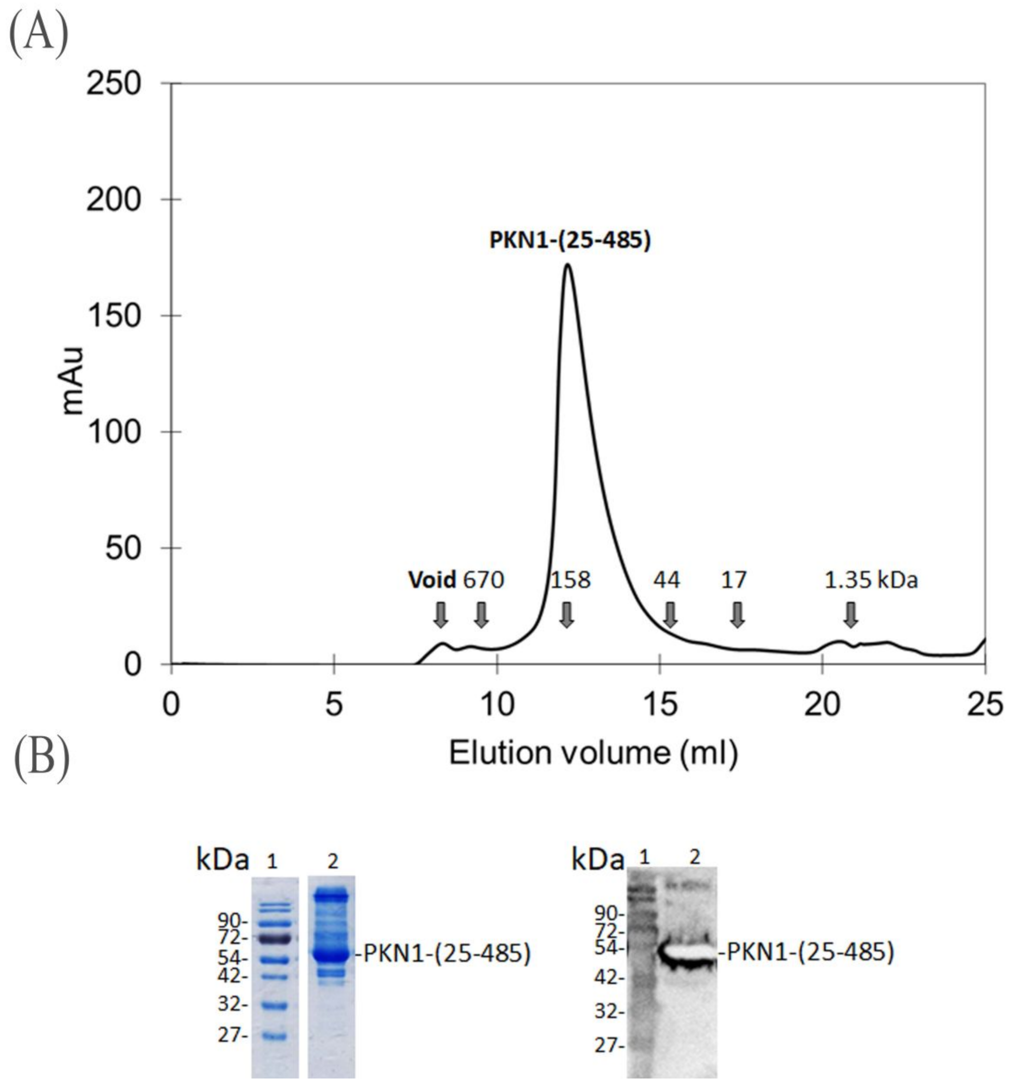

Figure S3. PKN1-(25-485) protein adopts a trimeric conformation under native conditions.

(A) Metal ion affinity-purified PKN1-(25-485) comprising the HR1 and C2 regions (Fig. 1(A)) was analyzed by Superdex-200 size exclusion chromatography in a buffer of 50 mM Tris-HCl pH7.5, 150 mM NaCl and 0.075% Brij-35. The protein markers used for calibration were: thyroglobulin (bovine), 670 kDa; γ-globulin (bovine), 158 kDa; ovalbumin (chicken), 44 kDa; myoglobin (horse), 17 kDa; and vitamin B12, 1.35 kDa (*Bio-Rad*). (B) The integrity and identity of recombinant PKN1-(25-485) were first resolved by 14% SDS-PAGE and subsequently confirmed by Coomassie blue staining and immunostaining using rabbit monoclonal anti-PKN1. Lane 1: protein size markers and lane 2: PKN1-(25-485). The estimated molecular weight for PKN1-(25-485) is 53.17 kDa.

1. Protein-lipid binding experiments
(CD and Trp fluorescence quenching analyses).

2. Empirical analyses of the molar stoichiometry of
protein (acceptor)-lipid (ligand) interactions
(Trp fluorescence quenching analyses).

3.Double reciprocal data analyses to estimate the
lipid-protein binding plateau $(F/F_o)^*$ value.

4. Calculation of the lipid-protein fractional saturation f_a.

5.Scatchard plot analysis for lipid-protein interaction.

6. Estimation of protein-lipid K_d value (s).

Figure S4. Experimental strategy for investigating PKN1 binding interactions with lipids.

The impact of lipid binding on the secondary or tertiary conformation of the target protein was determined by CD or intrinsic tryptophan fluorescence spectrometry. Protein-lipid binding curves (signals) were quantitatively analyzed to determine lipid-to-protein stoichiometry by means of intrinsic tryptophan fluorescence spectrometry. The plateau value of $(F/F_o)^*$ for lipid binding to protein was estimated by double reciprocal analysis. Fractional saturation (f_a) of lipid on the target protein was calculated based on the experimentally determined fractional saturation (F/F_o) value from Scatchard plot analysis to determine K_d and binding function **r** values (MATERIALS AND METHODS).

$$(C2)^{site\ I} + 3PL \leftrightarrow [(C2)^{siteI}\text{-}3PL],\ K_d(I) = \frac{[(C2)^{site\ I}][PL]^3}{[(C2)^{site\ I}\text{-}3PL]} \quad (1)$$

$$(C2)^{site\ II} + 6PL \leftrightarrow [(C2)^{site\ II}\text{-}6PL],\ K_d(II) = \frac{[(C2)^{site\ II}][PL]^3}{[(C2)^{site\ II}\text{-}3PL]} \quad (2)$$

$$(C2)^{site\ I}\text{-}3PL+6PL \leftrightarrow [6PL\text{-}^{site\ II}(C2)^{site\ I}\text{-}3PL],\ K_d(II) = \frac{[(C2)^{site\ I}\text{-}3PL][PL]^6}{[6PL\text{-}^{site\ II}(C2)^{site\ I}\text{-}3PL]} \quad (3)$$

$$^{site\ II}(C2)^{site\ I} + 9PL \leftrightarrow [6PL\text{-}^{site\ II}(C2)^{site\ I}\text{-}3PL] \quad (4)$$

Figure S5. Model describing the PKN1-(C2) interactions with cardiolipin or phosphatidic acid.

The conceptual model envisages that the dissociation constants for interactions of PKN1-(C2) (referred to as C2) with phospholipid (PL), K_d (I) and (II), can be expressed as equations (1) and (2), respectively. A variation is that binding to the type II low-affinity site occurred sequentially following initial formation of the $(C2)^{site\ I}$-3PL complex, as described by equation (3). Thus, the overall binding equation for the two-sites model can be described by equation (4). Either cardiolipin or phosphatidic acid interacted with one or both of the recombinant protein's distinct type I and type II PL binding sites, with the respective dissociation constants for these interactions defined as K_d (I) and K_d (II). [PL] represents the free phospholipid concentration available for either type I or type II PKN1-(C2) versus PL interactions.

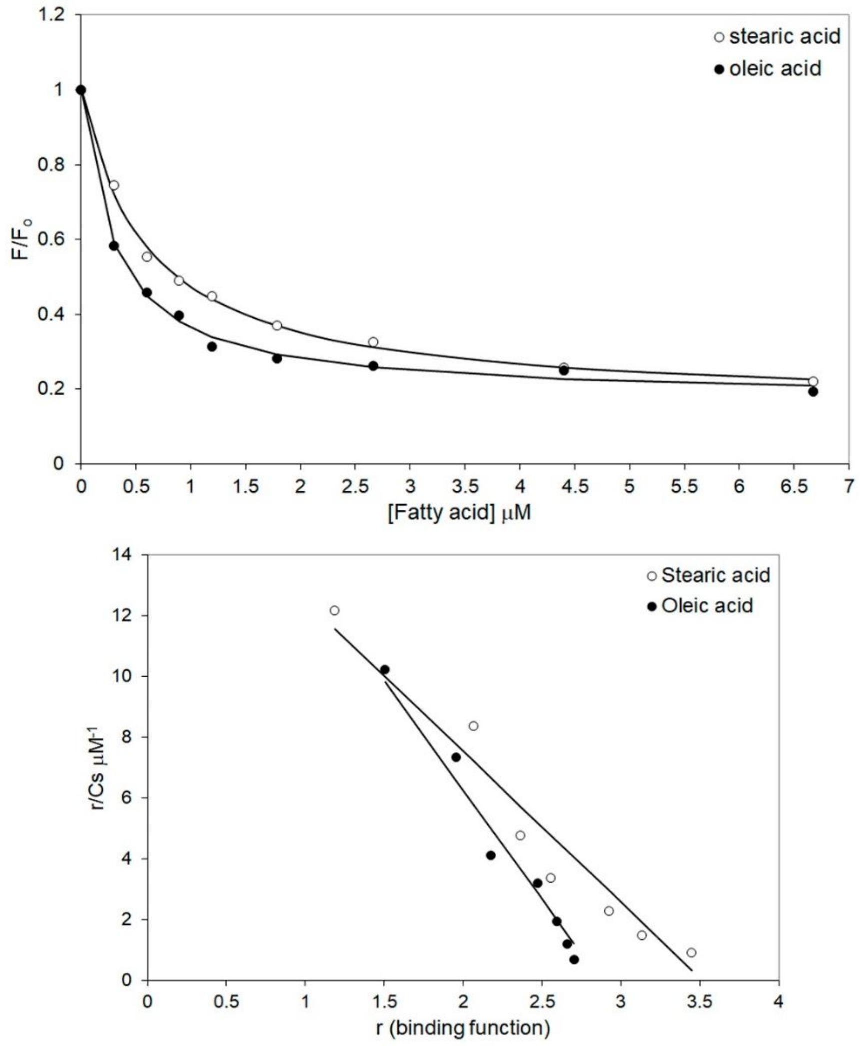

Figure S6. Binding curves for interactions of PKN1-(HR1) with fatty acids.
PKN1-(HR1) (0.17 μM) was titrated with either stearic acid or oleic acid in 20 mM phosphate buffer pH 7.4 and 25 mM NaCl at 25 ^{o}C. Fluorescence intensities at the emission wavelength of 340 nm were used to determine binding values. Molar ratios for lipid-to-protein interactions were determined as described in the legend of Fig. S4 (see also MATERIALS AND METHODS). The Scatchard plots were calculated based on the PKN1-Trp^{13} fluorescence quenching binding curves. The binding function (**r**) and free lipid concentration (***Cs***) were defined as described in the MATERIALS AND METHODS. The binding parameters are summarized in Table S3.
